# Influence of elevated temperature on bovine oviduct epithelial cells (BOECs)

**DOI:** 10.1371/journal.pone.0198843

**Published:** 2018-06-15

**Authors:** Łukasz Rąpała, Rafał R. Starzyński, Piotr Z. Trzeciak, Sebastian Dąbrowski, Małgorzata Gajewska, Piotr Jurka, Roman Smolarczyk, Anna M. Duszewska

**Affiliations:** 1 Division of Histology and Embryology, Department of Morphological Sciences, Faculty of Veterinary Medicine, Warsaw University of Life Sciences, Warsaw, Poland; 2 Polish Academy of Sciences, Institute of Genetics and Animal Breeding, Jastrzębiec, Poland; 3 Department of Physiological Sciences, Faculty of Veterinary Medicine, Warsaw University of Life Sciences, Warsaw, Poland; 4 Department of Small Animal Diseases with Clinic, Faculty of Veterinary Medicine, Warsaw University of Life Sciences, Warsaw, Poland; 5 Department of Gynecological Endocrinology, Faculty of Medicine, Medical University of Warsaw, Warsaw, Poland; University of Florida, UNITED STATES

## Abstract

The aim of this study was to evaluate the influence of elevated temperature on bovine oviduct epithelial cells (BOECs), based on the expression and localization of both heat shock protein 70 (HSP70), responsible for the cellular defence mechanism, and oviduct specific glycoprotein 1 (OVGP1) which is the most important embryotrophic protein. BOECs were cultured alone and co-cultured with cattle embryos at control (38.5°C) and elevated temperature (41°C) for 168 h. The elevated temperature had no effect on the viability of BOECs but exerted a negative effect on embryo development. The elevated temperature increased the expression of HSP70 and decreased the expression of OVGP1 at both mRNA and protein levels in BOECs cultured alone and those co-cultured with embryos. However, the presence of embryos limited the decrease in OVGP1 expression in BOECs at elevated temperature but did not alter the expression of HSP70. These results demonstrate for the first time the influence of elevated temperature on BOECs, consequently providing insights into the interactions between the embryo and the oviduct at elevated temperature.

## Introduction

Elevated ambient temperature inhibits the development of mammalian embryos [1] and cattle embryos are particularly vulnerable to high temperatures [2, 3, 4, 5, 6, 7].

*In vivo* and *in vitro* studies have demonstrated that heat stress can lead, among other things, to dysfunction in development of embryos and even to their death [5, 8, 9, 10]. However, in these studies the effect of high temperatures was not investigated in regard to oviductal functions. Therefore, this research focused on bovine oviduct epithelial cells (BOECs), which participate in the final maturation of gametes, fertilization and early embryo development [11, 12, 13, 14], and aimed to explore the response of these oviductal cells to elevated temperature.

BOECs lining the tunica mucosa of the oviduct comprise both ciliated and secretory cells [15]. The morphology of these cells undergoes dynamic changes depending on the estrous cycle [16]. BOECs secret many nutritional factors, such as amino acids, pyruvate [14], growth factors such as insulin-like growth factor alpha (IGF-α), fibroblast growth factor (FGF), and vascular endothelial growth factor (VEGF) [17, 18, 19], and antioxidants, as well as hypotaurine and taurine [20]. BOECs also secrete embryotrophic factors, among which the most important is oviduct specific glycoprotein 1 (OVGP1) that participates in fertilization and early embryo development [21]. Furthermore, BOECs secrete the tubal fluid wherein all processes related to gamete and embryo functions occur.

BOECs have been used in basic research to evaluate, among other things, the interactions between spermatozoa and epithelia and also the influence of ovarian steroid hormones on these cells [22, 23]. BOECs co-cultured with embryos are a major source of information on the molecular maternal-embryonic communication mechanisms [23]. BOECs have been used both in co-culture with cattle embryos as well as with embryos of other species [24, 25, 26], since BOECs do not exhibit species specificity. In all published research, BOECs had a positive effect on embryo development. This effect resulted not only from the secretion of growth and embryotrophic factors into the environment but also from the removal of toxic metabolites from the medium [22].

Therefore, in the present study a system comprising BOECs culture alone and co-culture with cattle embryos was used to analyse the influence of heat stress on these cells. BOECs were isolated at the time of ovulation, or a few days afterwards, because in this phase BOECs produce and secrete into the tubal fluid many factors and important proteins for the proper development of early embryos [27, 28]. In this *in vitro* experiment, the elevated temperature of 41°C was used because in outdoor cattle breeding this temperature had been measured *per rectum* during hot summer months in the USA [29].

The influence of elevated temperature on BOECs was evaluated based on the activation of heat shock protein 70 (HSP70), one of the most important proteins in cellular defensive mechanisms against thermal shock. This protein is mainly involved in the transport of proteins within the cell, the conferment of the proper conformation to newly created proteins and the restitution of native forms to denatured proteins. HSP70 is an anti-apoptotic protein both at physiological and elevated temperatures [30].

In addition, this study analyzed the effect of elevated temperatures on one of the most important embryotrophic proteins—oviduct specific glycoprotein 1 (OVGP1), which is secreted by BOECs. OVGP1 is an *exocrine* protein that, after release into the tubal fluid, participates in molecular changes in the zona pellucida leading to fertilization of the oocyte [31]. OVGP1 also prevents the phenomenon of polyspermy [32]. However, another essential role of OVGP1 lies in its embryotrophic properties [33, 34], because its presence helps the development of embryos both *in vivo* and *in vitro* [16, 35].

Hence, the objective of this research was to evaluate the influence of elevated temperature on bovine oviduct epithelial cells (BOECs), based on the expression and localization of HSP70 and OVGP1 in these cells.

## Materials and methods

### Ethics statement

All experiments and procedures were performed in compliance with the Polish Animal Welfare regulations and approved by the Local Ethics Commission for Animal Experiments of Warsaw University of Life Sciences.

### Reagents

The following reagents were used in the study: Hank’s medium (SIGMA, USA), gentamicin (SIGMA, USA), penicillin (SIGMA, USA), streptomycin (SIGMA, USA), PBS (SIGMA, USA), TCM-199 HEPES (SIGMA, USA), foetal bovine serum (FBS) (GIBCO, Scotland), mineral oil (SIGMA, USA), FSH (SIGMA, USA), β-oestradiol (SIGMA, USA), sodium pyruvate (MERCK, France), BSA fraction V (SIGMA, USA), BSA fraction FAF (SIGMA, USA), penicillamine (SIGMA, USA), hypotaurine (SIGMA, USA), epinephrine (SIGMA, USA), High Pure RNA Isolation Kit (Roche Diagnostics), SYBR Green (Roche Applied Science), Hybond-ECL nitrocellulose membranes (Amersham Biosciences), Trypan blue (SIGMA) antibody against HSP70 (Abcam, ab6535), antibody against OVGP1 (Abcam, ab74544), rabbit anti-mouse secondary antibodies (Santa Cruz Biotechnology, sc-2005), goat anti-rabbit secondary antibodies (Santa Cruz Biotechnology, sc-2004), ECL Plus (Amersham Life Sciences), Fluoromount (Sigma USA).

### Materials

Bovine oviducts (n = 60) and ovaries (n = 340) were collected *post mortem* from cattle slaughtered in Łmeat-Łuków, Meat Campany, Łuków, Poland’s facility in compliance with Polish Animal Welfare regulations. Oviducts were obtained from cattle in phase I of the estrous cycle (between 1^st^ and 4^th^ days, where day 1 represents ovulation) based on the ovarian morphology described by Ireland [36]. Selected oviducts were placed in Hank’s medium without Ca^2+^ and Mg^2+^, supplemented with antibiotics [50 μg/mL gentamicin, 100 IU/mL penicillin and 50 μg/mL streptomycin]. Ovaries were placed in PBS containing 0.2 mg/ml streptomycin and 250 IU/mL penicillin. Ovaries and oviducts were transported to the laboratory within less than 2 h at 30°C.

#### Preparation of BOECs

Bovine oviduct epithelial cells (BOECs) were mechanically isolated from the oviducts generally according to the procedure described by Rottmayer [16]. The end of the isthmus of each oviduct was placed in a 15 mL tube. 5 mL of Hanks medium without Ca^2+^ and Mg^2+^, supplemented with antibiotics [50 μg/mL gentamicin, 100 IU/mL penicillin and 50 μg/mL streptomycin] was syringed into the oviduct via the infundibulum, which was held in place with forceps. The oviduct was then gently squeezed in a stripping motion with forceps along the ampulla and isthmus to obtain BOECs. Then, BOECs were centrifuged 650xg for 10 min. and washed three times in TCM-199 HEPES medium without NaHCO_3_, supplemented with 10% FBS (vol/vol) and 50 μg/mL gentamicin, pH 7.4, and washed twice in cell culture medium (CM): TCM 199 supplemented with 10% FBS (vol/vol) and 100 IU/mL penicillin and 50 μg/mL streptomycin, adjusted to pH 7.4. The cells were cultured for 48 h to form aggregates of BOECs in a 5% CO_2_ incubator at 38.5°C. Then, BOEC aggregates were selected and washed three times in CM medium.

#### Embryo sources

Bovine embryos were obtained according to the procedure commonly used in our laboratory [37]. Immature cumulus-oocyte complexes (COCs) were aspirated from ovarian follicles with a diameter of 2–6 mm and washed twice in the following medium: TCM 199 HEPES without NaHCO_3_ supplemented with 10% FBS (vol/vol) and 50 μg/mL gentamicin, pH 7.4. Groups of 20 COCs containing immature oocytes were placed in 500 μl drops of oocyte maturation medium [TCM 199 with HEPES containing 10% FBS (vol/vol) and 0.02 IU NIH-pFSH/mL, 1 μg/mLβ-oestradiol and 0.2 mM sodium pyruvate, 50 μg/mL gentamicin, pH 7.4] and cultured in a 5% CO_2_ incubator (HERAEUS, USA) for 24 h at 38.5°C.

The semen from Jersey bull was frozen in straws. The semen straws were thawed at 37°C, centrifuged (200xg) for 10 min. and washed in 2 mL of Sp-TALP containing 6 mg/mL BSA fraction V and 50 μg/mL gentamicin, pH 7.4. Spermatozoa underwent capacitation by the swim-up method [38] in 1mL of Sp-TALP. *In vitro* fertilization was performed in Fert-TALP containing 6 mg/mL BSA fraction FAF, 0.2 mM sodium pyruvate, PHE (20 μM penicillamine, 10 μM hypotaurine, 1 μM epinephrine), 50 μg/ml gentamicin and 2 μg/mL heparin, pH 7.4. Co-incubation of oocytes and spermatozoa was conducted at 38.5°C in a humidified atmosphere with 5% CO_2_ for 18h.

After 18 hours post fertilization (hpf), putative zygotes were denuded of cumulus cells by vortexing in CM at 300g for 3 min. Zygotes with normal morphology (with regular shape, appearance of perivitelline space with 1^st^ and 2^nd^ polar bodies, homogeneous cytoplasm without any fragmentation or degeneration) were randomly assigned to co-culture with BOECs.

### Experimental design

Aggregates of BOECs were cultured alone (variant I) or co-cultured with cattle embryos (variant II) at the control temperature (38.5°C) and the experimental temperature (41°C) for 168 h (7 days).

During the embryo co-culture their development was evaluated after 48 hpf (theoretically the time at which embryos should have reached the 2–4 cell stage) and after 168 hpf (theoretically the time at which embryos should have reached the blastocyst stage).

After 168 h, BOECs were removed from both variants and a portion of the BOECs was used for viability assays, whereas the remaining cells from both culture variants were frozen at -70°C for prolonged storage up to two weeks. After thawing BOECs from both culture variants, analysis of *hsp70* and *ovgp1* gene expressions at the mRNA level (Real Time PCR) and protein level (Western Blot) was carried out. In addition, localization of HSP70 and OVGP1 proteins in BOECs from both variants was determined by immunofluorescent staining.

### BOECs culture and co-culture with cattle embryos

Groups of approximately 20 BOEC aggregates were cultured alone or co-cultured with 20 zygotes in 40 μl droplets of TCM199 supplemented with 10% (vol/vol) FBS and 50 μg/mL gentamicin, pH 7.4, overlaid with mineral oil. BOECs were cultured (variant I) or co-cultured with embryos (variant II) at control (38.5°C) and elevated (41°C) temperatures in 5% CO_2_ incubators (HERAEUS, Smart Cell—HEAL FORCE, USA) for 168 h. After 48 and 144 hpf the 20 μl medium was replaced with fresh medium.

#### Analysis of cattle embryo development

Development of embryos co-cultured with BOECs (variant II) at the control temperature of 38.5°C (n = 920) and elevated temperature of 41°C (n = 920) was evaluated after 48 hpf (cleavage stage) and again after 168 hpf (blastocyst stage). The rate of development at different stages was calculated from the number of zygotes.

### Estimation of BOEC viability

After 168 hpf, cell viability of BOEC aggregates was estimated by the Trypan blue assay. Aggregates were stained with 0.2% trypan blue at room temperature. The experiment was replicated 10 times with a total of 100 aggregates from each of the control (38.5°C) and elevated (41°C) temperature groups. The viability of BOECs was determined by light microscopy using a MicroImage Olympus Optical Co. (GMBH, Europe).

### Quantitative Real-Time PCR analysis of *hsp70* and *ovgp1* gene expression in BOECs

Total RNA from BOECs cultured (variant I) or co-cultured with embryos (variant II) at control (38.5°C) and elevated (41°C) temperatures was extracted using the High Pure RNA Isolation Kit (Roche Diagnostics) according to the manufacturer’s instructions. RNA concentrations were assessed using NanoDrop (ND-100). Oligonucleotide primer sequences for *hsp70* and *ovgp1* were designed using the Primer Blast NCBI program (Table 1). Detection of amplified products was carried out using the SYBR Green method [39]. To confirm amplification specificity, PCR products from each primer pair were subjected to melting curve analysis and subsequent agarose gel electrophoresis. For data analysis, Light Cycler 3.5 Software was used. Transcript levels were calculated relative to transcription of the housekeeping genes *S18 and H2A* in each sample.

**Table 1:** Oligonucleotide primers used in Real Time PCR assays.

Gene	GenBank accesion no.	Primer sequence	Product size (bp)
HSP70	AY149618.1	*for* 5’-CAGTCTGCTGATGATGGGGTTA-3’ *rev* 5’-GACAAGTGCCAGGAGGTGATTT-3’	117
OVGP1	NM_001080216.1	*for* 5’-GGAAGTACACCAAGCAAGCTG-3’ *rev* 5’-AGGTCCAATGTCCACACCAT-3’	214
S18	NM_001033614	*for* 5’-GAGGATGAGGTGGAACGTGT-3’ *rev* 5’-CCTGTGGTCTTGGTCTGCT-3’	239
H2A	NM_174809	*for* 5’-AGGACGACTAGCCATGGACGTGTG-3’ *rev* 5’-CCACCACCAGCAATTGTAGCCTTG-3’	209

The expression of *hsp70* mRNA was evaluated in BOECs cultured alone (variant I) at control (n = 10) and elevated temperatures (n = 10) and co-cultured with embryos (variant II) at control (n = 9) and elevated temperatures (n = 6). The expression of *ovgp1* mRNA was evaluated in BOECs cultured alone or co-cultured with embryos at control and elevated temperatures (n = 9 for each of the 4 groups)

### Western blot analysis of HSP70 and OVGP1 proteins in BOECs

Total extracts of BOECs cultured (variant I) or co-cultured with embryos (variant II) at control (38.5°C) and elevated (41°C) temperatures were isolated. Thirty micrograms of BOEC total extracts (for HSP70 and OVGP1 detection) were resolved on 9% (for HSP70 detection) or 6% (for OVGP1 detection) SDS-polyacrylamide gels and transferred to Hybond-ECL nitrocellulose membranes. The membranes were initially blocked by gentle agitation in Tween 20-Tris-buffered saline containing 5% fat-free dry milk at room temperature for 1 h followed by overnight incubation at 4°C with mouse monoclonal antibody raised against HSP70 (concentration 1:2000). Monoclonal Anti-Heat Shock Protein 70 antibody (mouse IgG1 isotype) is derived from the BRM-22 hybridoma produced by the fusion of mouse myeloma cells and splenocytes from BALB/c mice immunized with purified native full length bovine brain HSP70. For OVGP1 detection the rabbit polyclonal antibody raised against OVGP1 (concentration 1:1000) was used. An 18 amino acid synthetic peptide from near the N terminus of human OVGP1 (NP_002548) has been used as an immunogen. Membranes were then washed and incubated with peroxidase-conjugated rabbit anti-mouse or goat anti-rabbit secondary antibodies (concentration 1:20000) for 1 hat room temperature. Immunoreactive bands were detected using the enhanced chemiluminescence Western blot detection ECL Plus system (Pierce^™^ ECL Plus Western Blotting Substrate, Thermo Fisher Scientific).

The expression of HSP70 and OVGP1 proteins in BOECs cultured alone (variant I) or co-cultured with embryos (variant II) at control and elevated temperatures (n = 3 for each of the 4 groups) was evaluated based on densitometric analysis using Biorad Molecular Imager FX and Quantity One software.

#### Immunolocalization of HSP70 and OVGP1 in BOECs

BOECs cultured alone (variant I) or co-cultured with embryos (variant II) at control (38.5°C) and elevated (41°C) temperatures were washed twice in PBS. Then, BOECs from each condition and temperature were fixed for 20 min in 4% (v/v) formaldehyde. After fixation and permeabilization in 0.5% Triton X(PBS-X) for 20 min, BOECs were washed twice for 10 min in PBS, blocked for 1 h at room temperature with 5% normal goat serum, and incubated with a mouse monoclonal antibody raised against HSP70 (concentration 1:100), or rabbit polyclonal antibody raised against OVGP1 (concentration 1:100) at 4°C for 18 h. Next, samples were washed and incubated with rabbit anti-mouse or goat anti-rabbit Alexa Fluor 488-conjugated secondary antibodies, respectively, (concentration 1:1000) for 1h at room temperature. Nuclei were counterstained with Hoechst 33342. Samples used as negative controls were stained only with secondary antibodies conjugated with Alexa Fluor 488 dye and counterstained with Hoechst 33342. All slides were mounted with Fluoromount (Sigma Aldrich F4680, USA). Analyses were carried out under a IX70 FV 500 confocal microscope (Olympus).

The immunofluorescence localizations of HSP70 and OVGP1 proteins in both BOECs variants at control (n = 5) and elevated (n = 5) temperatures and BOECs co-cultured with embryos at control (n = 5) and elevated (n = 5) temperatures were evaluated based on densitometry analysis using MicroImage software Olympus Optical CO. (Europa) GMBH.

### Statistical analysis

Statistical analysis was performed using Statgraphics Plus 5.1 software (Manugistics Inc., USA). The estimation of BOECs viability was analysed using the F-test. The effect of control and elevated temperatures on: 1) embryo development, 2) expression of *hsp70* and *ovgp1* mRNA in both BOECs culture variants, 3) expression of HSP70 and OVGP1 proteins in both BOECs culture variants, 4) immunolocalization of HSP70 and OVGP1 proteins in both BOECs culture variants, was determined using one-way ANOVA and multiple pair-wise comparisons using the LSD test. Differences at P<0.05 were considered significant and P<0.01 were considered highly significant.

## Results

### Effect of elevated temperature on development of embryos co-cultured with BOECs

**Fig 1:**
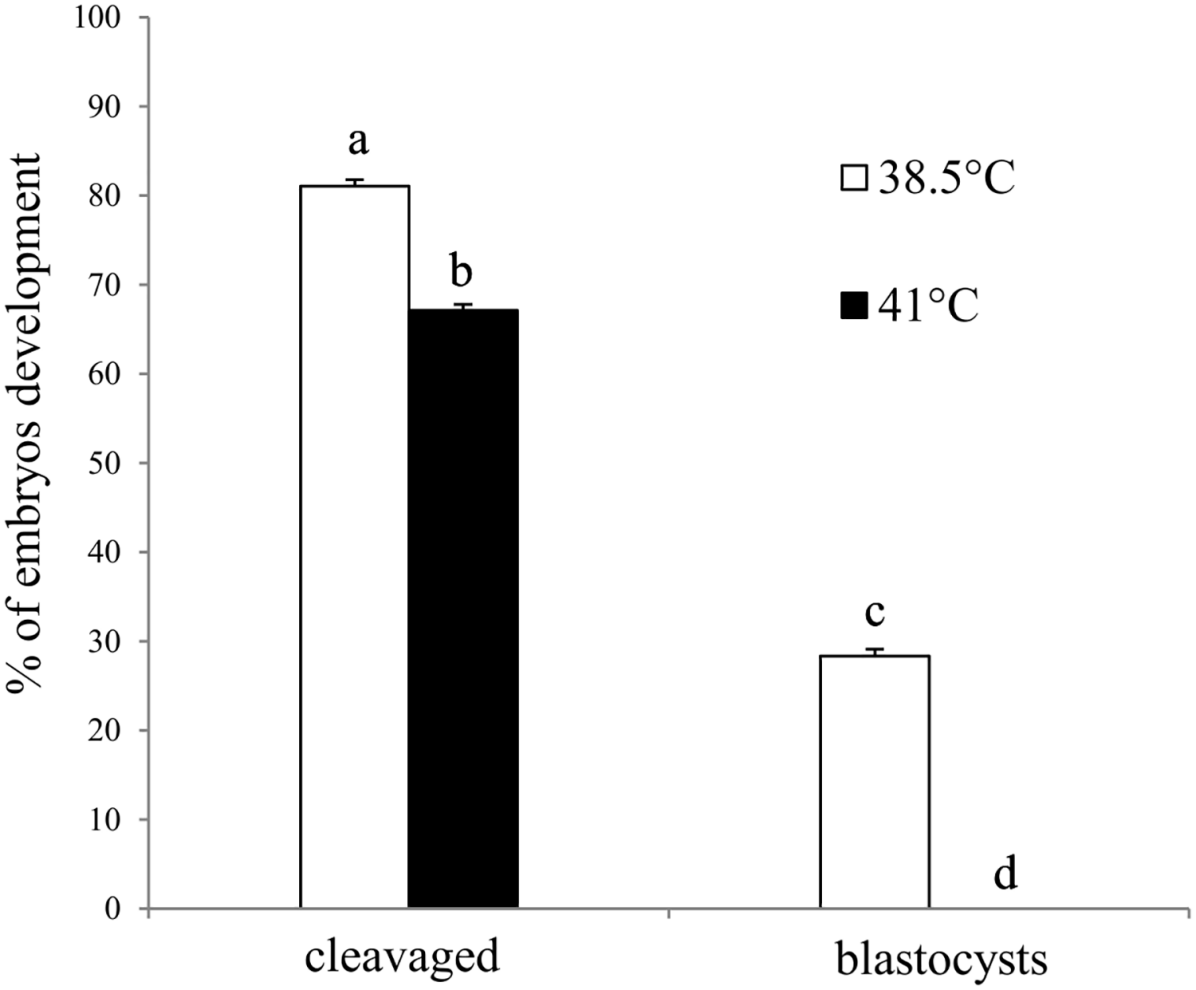
Analysis of development of cattle embryos co-cultured with BOECs at control (38.5°C) and elevated (41°C) temperatures for 168 h post fertilization. Data presented as Mean±SEM. Bars with different letters denote statistical differences at P<0.01.

Analysis of development of embryos co-cultured with BOECs (variant II) at control temperature (38.5°C) and elevated temperature (41°C) is shown in Fig 1 (S1 File).

The embryos co-cultured with BOECs developed better at the control temperature than at elevated temperature, as evidenced by a significantly higher percentage of embryos at the cleaved stage after 48 hpf and blastocysts after 168 hpf (P<0.01).

Culture of BOECs with embryos at elevated temperature reduced the number of two- and four-cell embryos (% of cleavage), and arrested further embryonic development (% of blastocysts). None of the embryos co-cultured with BOECs at 41°C reached the blastocyst stage, in contrast to the control temperature, where a high percentage of embryos achieved the blastocyst stage (28.37±0.78).

### Effect of elevated temperature on BOECs viability

The elevated temperature had no effect on BOECs viability. The percentage of viable cells was similar in BOECs cultured at control (38.5°C) and elevated (41°C) temperatures (86.31±1.16 vs 85.62±2.18) (S2 File).

### Effect of elevated temperature on HSP70 expression and its immunolocalization in BOECs

**Fig 2:**
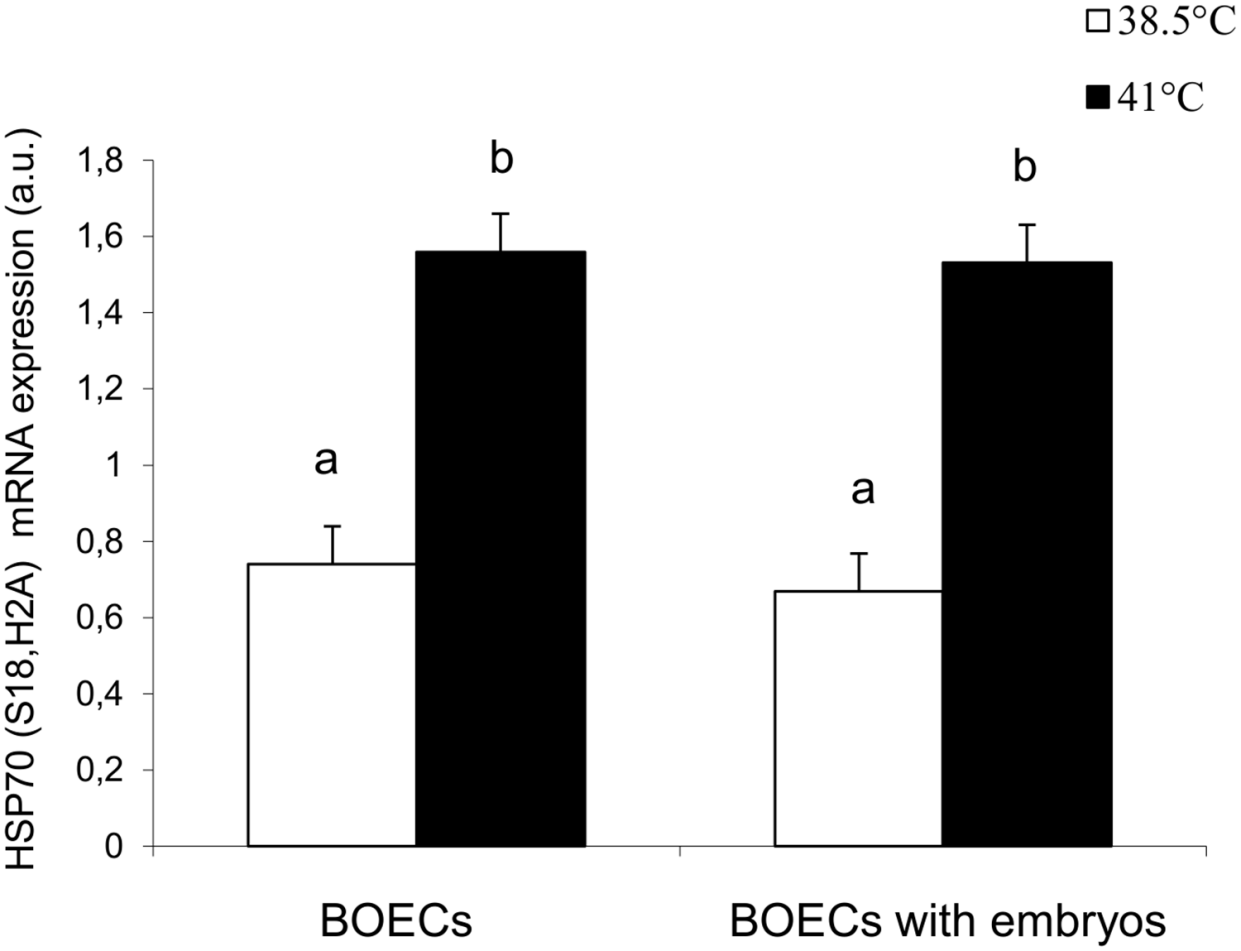
Expression of *hsp70* mRNA in BOECs cultured alone and co-cultured with cattle embryos at control (38.5°C) and elevated (41°C) temperatures for 168 h post fertilization. Data presented as Mean±SEM. Bars with different letters denote statistical differences at P<0.05.

Expression of *hsp70* in BOECs cultured alone (variant I) and co-cultured with cattle embryos (variant II) in control and elevated temperatures is shown in Fig 2 (S3 File). In both variants, the expression of *hsp70* in BOECs exposed to elevated temperature (41°C) was significantly higher than in control conditions (P<0.01). There was no difference in the expression of *hsp70* between the two variants of the culture at control and elevated temperatures.

**Fig 3:**
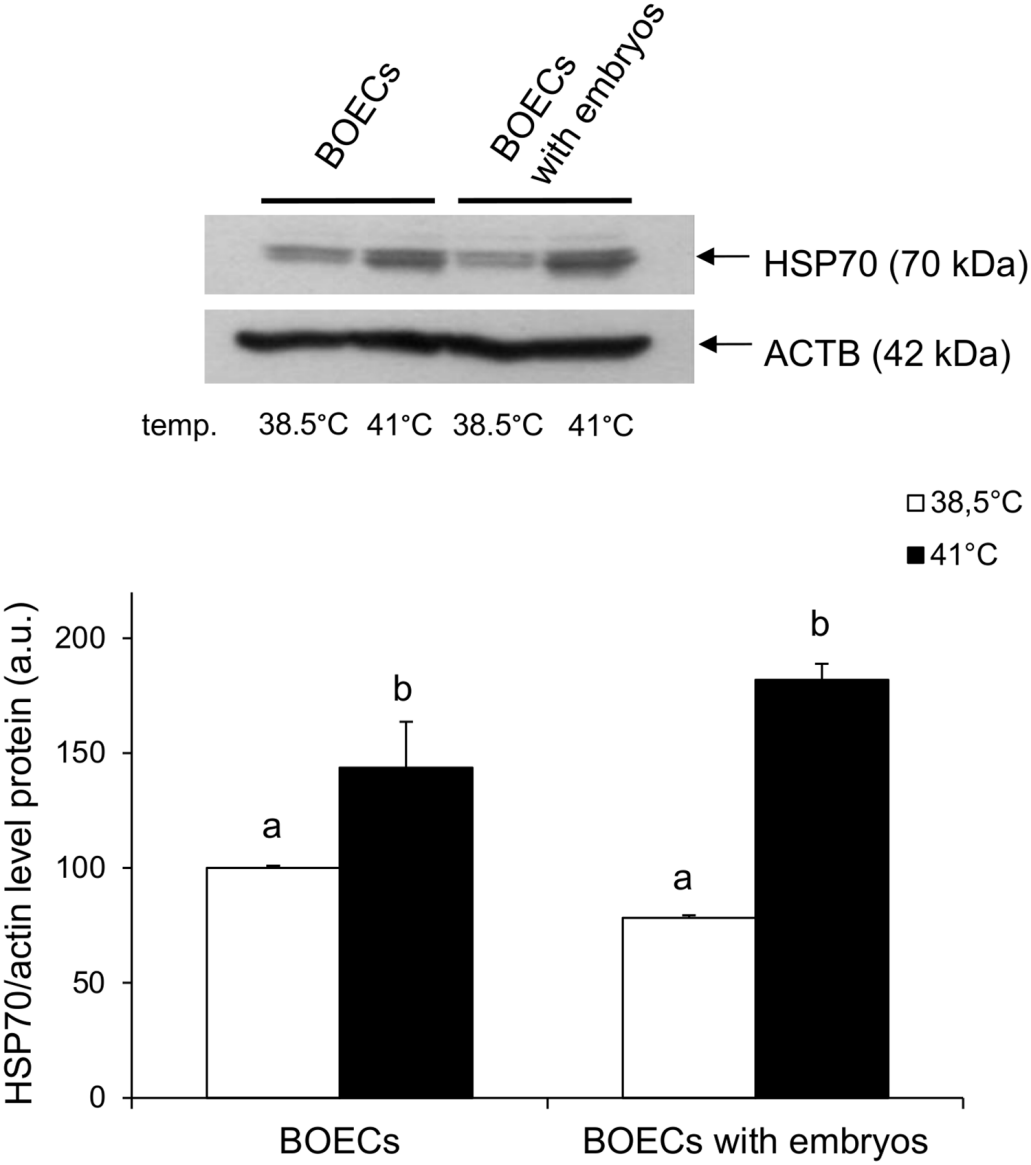
Analysis of HSP70 in BOECs cultured alone and co-cultured with cattle embryos at control (38.5°C) and elevated (41°C) temperatures for 168 h post fertilization. A) Western blots of HSP70 protein. B) Densitometric quantification of HSP70 protein expression. Data presented as Mean±SEM. Bars with different letters denote statistical differences at P<0.05.

**Fig 4:**
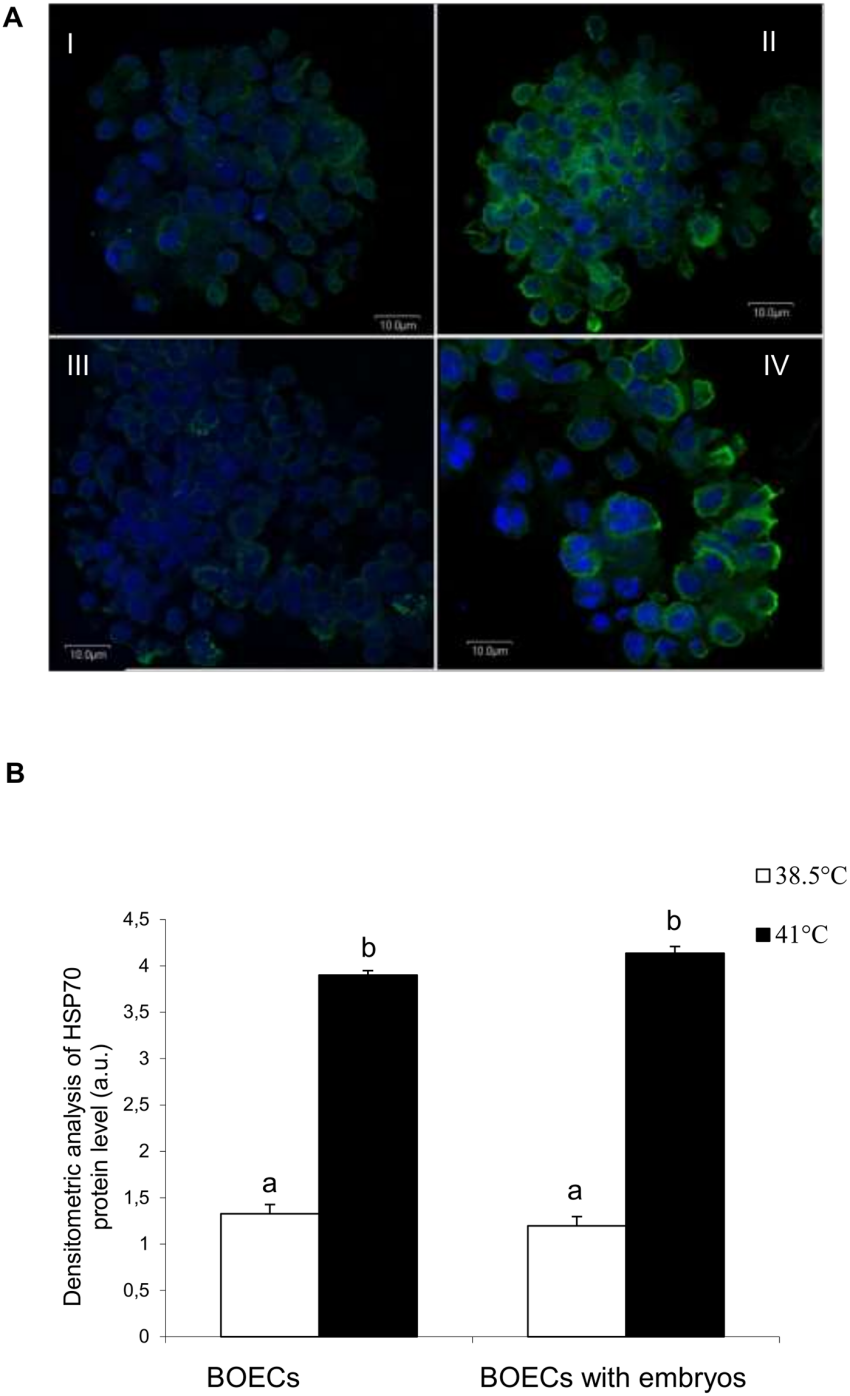
Fluorescent analysis of HSP70 in BOECs cultured alone and co-cultured with cattle embryos at control (38.5°C) and elevated (41°C) temperatures for 168 h post fertilization. A) Representative images of immunofluorescent staining of HSP70 in BOECs cultured alone at control (38.5°C) (I) and elevated (41°C) (II) temperatures, or co-cultured with cattle embryos at control (38.5°C) (III) and elevated (41°C) (IV) temperatures. Nuclei were counterstained with Hoechst 33342 (blue); HSP70 localization was determined using antibody against HSP70 and Alexa Fluor 488-conjugated secondary antibody (green). B) The IOD values of HSP70-related green fluorescence were measured using the MicroImage Olympus Optical analysis system. The data are presented as Mean±SEM. Bars with different letters denote statistical differences at P<0.05.

Western blot analysis revealed increased levels of HSP70 protein in both BOECs culture variants at elevated temperature in comparison to the control temperature (P<0.05) (Fig 3) (S3 File). However, there was no difference in the expression of HSP70 between the two variants of BOECs culture carried out at the same temperature. Immunofluorescent staining demonstrated that HSP70 protein was localized in the cytoplasm of BOECs cultured alone and those co-cultured with embryos at both temperatures, 38.5°C and 41°C (Fig 4) (S3 File). Densitometric analysis of the immunofluorescent staining confirmed the Western blot results, showing that at elevated temperature the level of HSP70 in both BOECs culture variants was significantly higher than at the control temperature (P<0.05). No difference in the expression of HSP70 was noted between both variants of BOECs culture at the control and elevated temperatures.

### Effect of elevated temperature on OVGP1 expression and immunolocalization in BOECs

**Fig 5:**
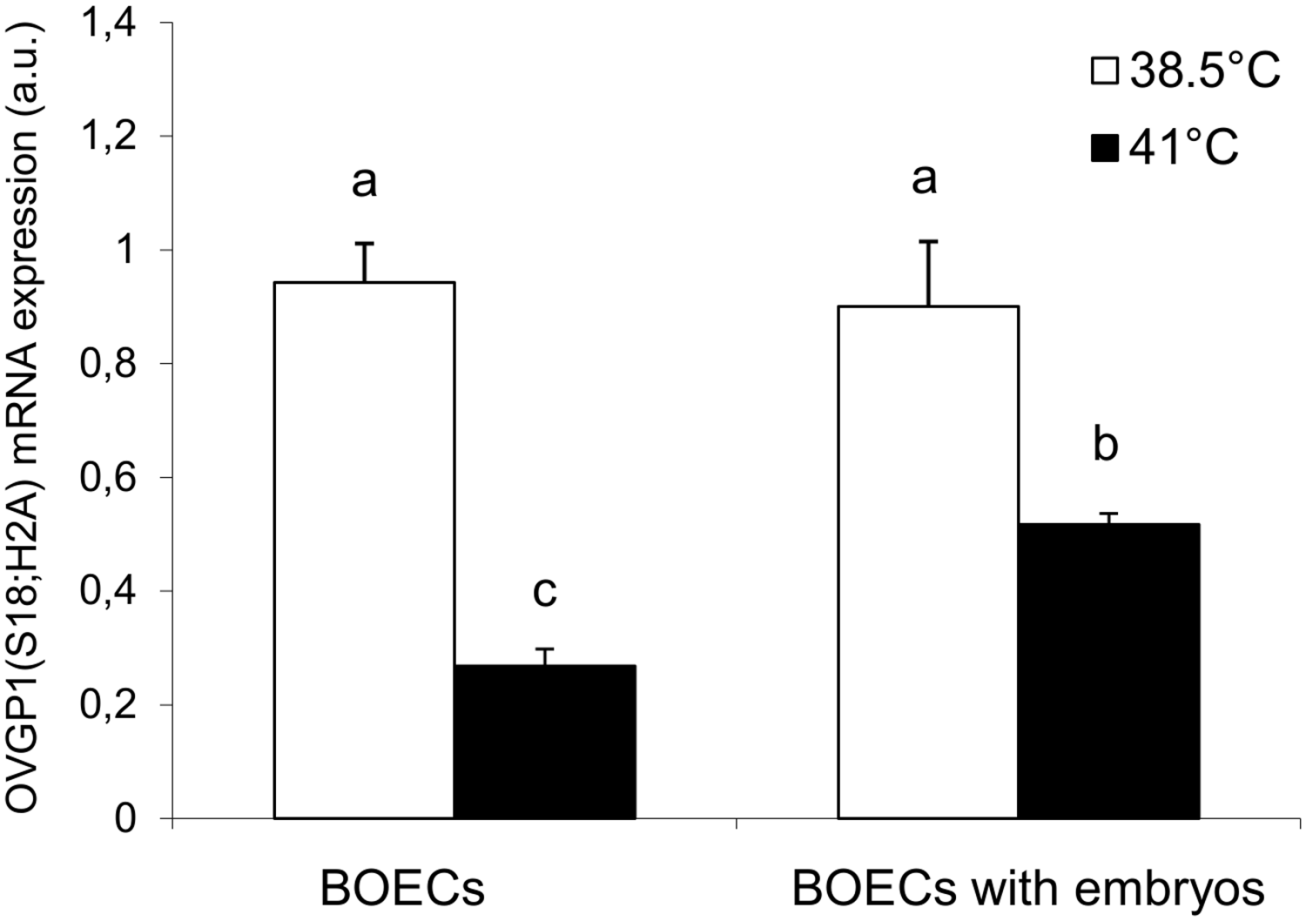
The expression of ovgp1 mRNA in BOECs cultured alone and co-cultured with cattle embryos at control (38.5°C) and elevated (41°C) temperatures for 168 h post fertilization. Data presented as Mean±SEM. Bars with different letters denote statistical differences at P<0.01.

Results of *ovgp1* gene expression analysis in BOECs cultured alone (variant I) and co-cultured with cattle embryos (variant II) at control (38.5°C) and elevated (41°C) temperatures is presented in Fig 5 (S4 File). In BOECs cultured alone or co-cultured with embryos the expression of *ovgp1* at elevated temperature was significantly lower than under the control conditions (P<0.01). At the control temperature, there was no difference in the expression of *ovgp1* between both variants of BOECs culture but at the elevated temperature a difference between both culture variants was observed (P<0.01).

**Fig 6:**
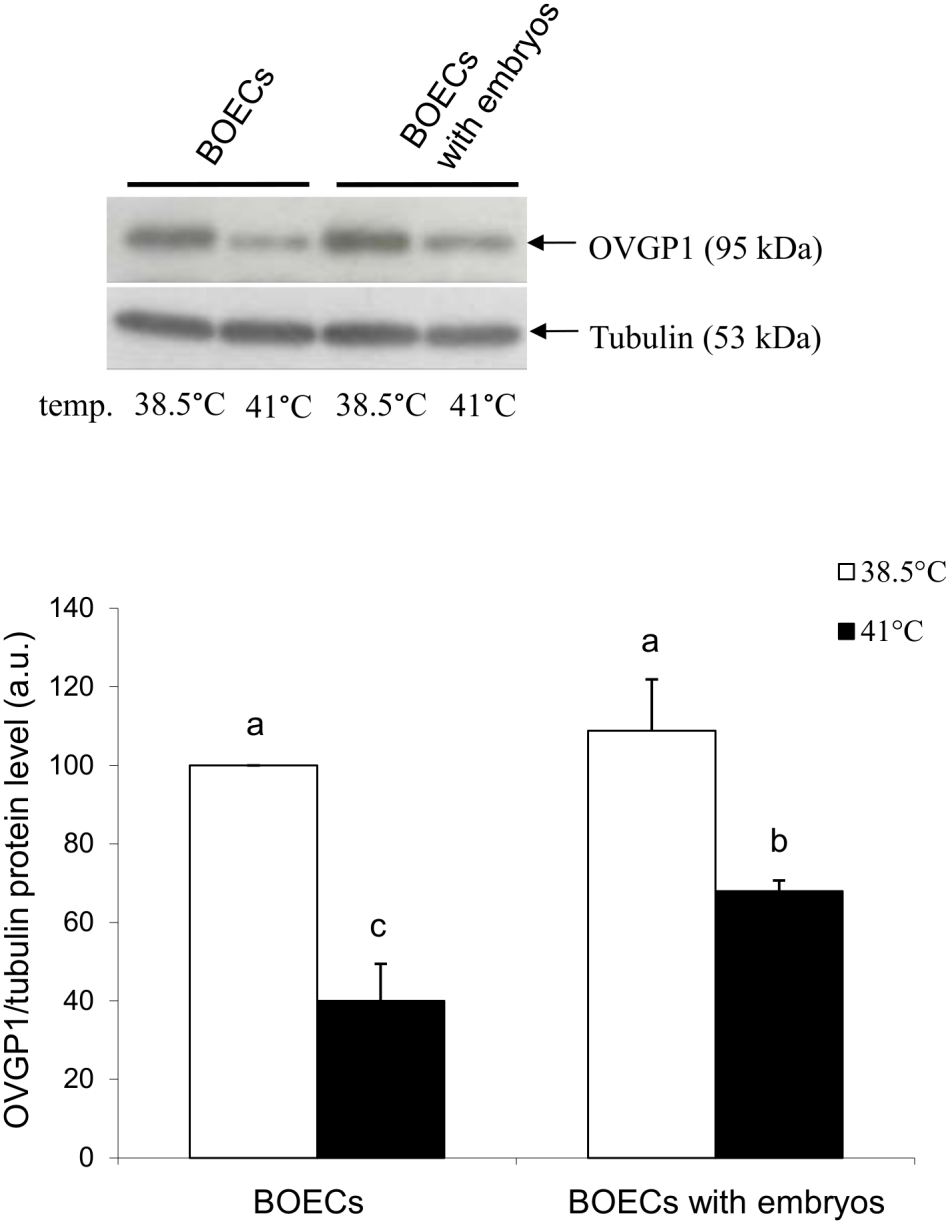
Analysis of OVGP1 in BOECs cultured alone and co-cultured with cattle embryos at control (38.5°C) and elevated (41°C) temperatures for 168 h post fertilization. A) Western Blot analysis of OVGP1 protein. B) Densitometric quantification of OVGP1 protein. Data presented as Mean±SEM. Bars with different letters denote statistical differences at P<0.05.

Western blot analysis revealed a decreased OVGP1 protein level in BOECs cultured alone at elevated temperature when compared to the control temperature (P<0.05) (Fig 6) (S4 File). Furthermore, in BOECs co-cultured with embryos the level of OVGP1 was significantly lower at the elevated temperature than under the control conditions (P<0.05). There was no difference in the expression of OVGP1 protein between both variants at the control temperature but there was a difference in the OVGP1 protein level between both variants of culture at the elevated temperature (P<0.05).

**Fig 7:**
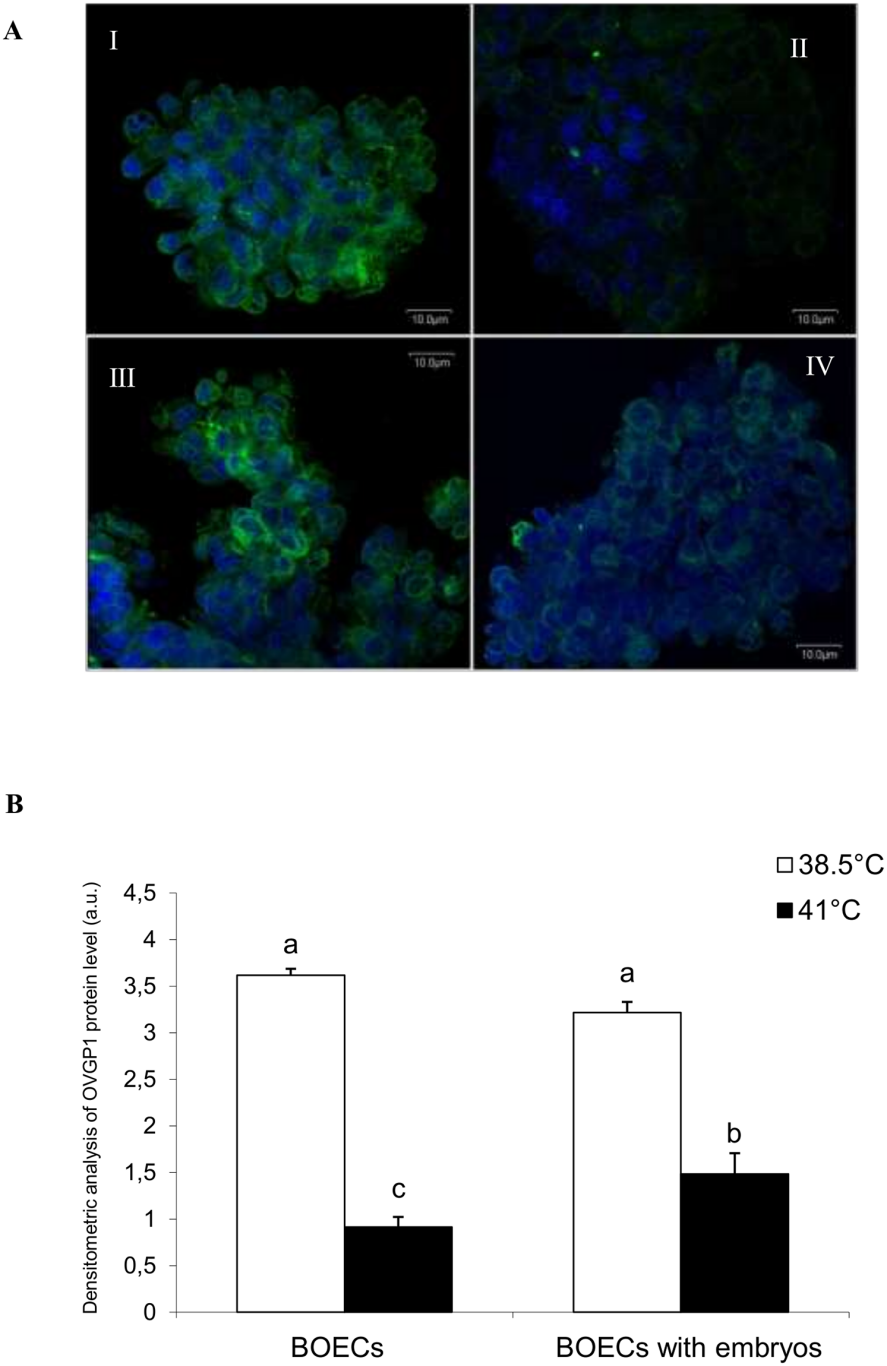
Fluorescent analysis of OVGP1 in BOECs cultured alone and co-cultured with cattle embryos at control (38.5°C) and elevated (41°C) temperatures for 168 h post fertilization. A) Representative images of immunofluorescent staining of OVGP1 in BOECs cultured alone at control (38.5°C) (I) and elevated (41°C) (II) temperatures, or co-cultured with cattle embryos at control (38.5°C) (III) and elevated (41°C) (IV) temperatures. Nuclei were counterstained with Hoechst 33342 (blue); OVGP1 localization was determined using antibody against OVGP1 and Alexa Fluor 488-conjugated secondary antibody (green). B) The IOD values of OVGP1-related green fluorescence were measured using the MicroImage Olympus Optical analysis system. The data are presented as Mean±SEM. Bars with different letters denote statistical differences at P<0.05.

Immunofluorescent staining revealed the cytoplasmic localization of OVGP1 in BOECs cultured alone (variant I) and those co-cultured with embryos (variant II) at control and elevated temperatures (Fig 7) (S4 File). Fluorescence intensity confirmed the Western blot results (Fig 6). Representative images shown in Fig 7A as well as densitometric analysis demonstrate that in both variants of cell culture the level of OVGP1 was significantly lower at the higher temperature than in the control conditions (P<0.05). There was no difference in the expression of OVGP1 protein between both variants of cell culture at the control temperature However, there was a slight increase in cytoplasmic OVGP1 protein expression in BOECs co-cultured with embryos at 41°C when compared with BOECs cultured alone at the higher temperature. Densitometric analysis revealed that these results were statistically significant (P<0.05) (Fig 7).

## Discussion

The action of heat stress on somatic cells and embryos is a well recognized phenomenon, which can lead to disorders in the proliferation and differentiation of cells, negative nuclear and cytoplasmic changes, and disintegration of the cytoskeleton causing the displacement of intracellular organelles. Furthermore, heat stress damages cell nuclei, leading to aggregation of proteins and functional and structural changes in the cell membrane [40, 41]. Heat stress induces apoptosis of both somatic cells and embryos [42, 43].

In the present study, permanent heat stress had no negative impact on the survival of BOECs. In contrast to BOECs, embryos at early stages were thermosensitive, which was demonstrated by inhibition of their development at elevated temperature. Similar observations were made when embryos were cultured in synthetic media at elevated temperatures [3, 5, 9, 10, 41, 44, 45, 46] and in our previous study in which the embryos were co-cultured with BOECs [47].

The maintenance of vitality of BOECs at elevated temperature is connected with activation of the molecular defence mechanisms. HSP70 plays a crucial role in this phenomenon and its presence is observed in many cell types, including embryos [48]. Also in this research, the expression of this protein was observed in BOECs at the control temperature. However, elevated temperature increased expression of the *hsp70* gene in BOECs. Similar results were obtained in experiments conducted on skeletal muscles, heart and liver cells [49]. Increased expression of the *hsp70* gene in BOECs cultured alone and in those co-cultured with embryos may be caused by activation of HSF1, which is physiologically associated with HSP70, blocking its activity. Increased temperature induces dissociation of the HSP70-HSF1 complex, allowing trimerization of released HSF1 monomers and binding to the promoter region of *HSP* genes containing the HSE (heat shock element) sequence. This in turn activates the transcription of *HSPs* [50, 51].

In the present study, an increase in the expression of the *hsp70* gene in BOECs cultured and co-cultured with embryos at elevated temperature was also confirmed at the protein level. This could result from both increased transcriptional activity of *HSP70* mRNA in BOECs, and from promotion of HSP70 mRNA in cells during heat stress. The additional phenomenon augmenting the translation of mRNA could be “leakage” of the ribosomes resulting from the purposeful omission of the inhibitor sequences located at the 5’ end of mRNA [52].

In BOECs, the HSP70 protein is located in the cytoplasm, and an increase in its expression at elevated temperature was evidenced by the strong fluorescent signal of HSP70 staining in BOECs cultured alone and in those co-cultured with embryos. It is worth emphasising that the presence of embryos did not influence the level of HSP70 in BOECs at the control or the elevated temperatures. Data reported by others support these findings, demonstrating that early embryos stimulate secretory cells to release nutritional factors, among other compounds, consequently creating optimal conditions for their development [14]. This is an interesting result considering the fact that in our earlier research we found that BOECs stimulated embryos to switch on their defence mechanism, based on finding HSP70 at significantly higher levels than in embryos cultured in the synthetic medium: KSOMaa—potassium simplex optimised medium modified by amino acids [47].

Despite switching on the defence mechanisms in BOECs, elevated temperature caused negative changes in these cells, which may influence the formation and early development of embryos. Heat stress led to a decrease in the level of OVGP1 in BOECs. Several other studies have analysed the expression of OVGP1 in different phases of the reproductive cycle, however, these studies were carried out under physiological temperature [16, 53]. The present research showed that elevated temperature led to decreased OVGP1 gene and protein expression in BOECs cultured alone and in those co-cultured with cattle embryos.

The reduction in OVGP1 in BOECs at elevated temperature was also demonstrated at the protein level. A major cause of this was the decreased expression of *OVGP1* gene leading to decreased level of OVGP1 protein. Downregulated OVGP1 expression might have resulted from disruption of the splicing process, whereby the creation of matured transcripts is stopped [54]. During heat stress, this disruption also suppresses the activity of translational factors: eIF4E and eIF2 recognize the "skullcap” formed at the 5' end of mRNA *hsp70*, which plays the main role in controlling the initiation of translation [52].

The OVGP1 protein occurs in the cytoplasm of BOECs. According to some studies, OVGP1 appears not only in intracellular droplets but also in lysosomes and endosomes [32, 33]. After its release into the oviduct fluid, OVGP1 can bind to both oocytes and spermatozoa [35]. At elevated temperature, the expression of OVGP1 was reduced. This indicates that elevated temperatures may negatively influence the reproductive processes by, among others, decreasing the levels of OVPG1.

It is interesting to note that at elevated temperature the presence of embryos limited the decline in OVGP1 expression in BOECs at the mRNA and protein levels. At elevated temperature, when the processes of transcription and translation is inhibited in cells, the additional signal emanating from embryos may stimulate BOECs to increase expression of this protein in order to support their development. Further studies are needed to determine the exact mechanisms of these molecular interactions.

## Conclusions

Elevated temperature had a negative influence on both embryo development and bovine oviduct epithelial cells (BOECs). Although elevated temperature did not influence survival of BOECs, it led to changes in the expression of HSP70 and OVGP1 genes in BOECs on both the mRNA and protein levels. At elevated temperature, the defence mechanism in BOECs may first be observed based on the increase of HSP70 expression. The presence of embryos did not influence expression of this protein in BOECs. At elevated temperature, the expression of one of the most important embryotrophic glycoproteins, OVGP1, was reduced on both the mRNA and protein levels. Decreased OVGP1 expression in BOECs may intensify the negative consequences of thermal stress on embryo development, increasing their thermosensitivity.

## Supporting information

S1 File Effect of elevated temperature on development of embryos co-cultured with BOECs. (XLSX)

S2 File Effect of elevated temperature on BOECs viability. (XLSX)

S3 File Effect of elevated temperature on HSP70 expression and its immunolocalization in BOECs cultured alone and co-cultured with cattle embryos. (XLSX)

S4 File Effect of elevated temperature on OVGP1 expression and its immunolocalization in BOECs cultured alone and co-cultured with cattle embryos. (XLSX)
